# Trunk rotation and hip joint range of rotation in adolescent girls with idiopathic scoliosis: does the "dinner plate" turn asymmetrically ?

**DOI:** 10.1186/1748-7161-3-1

**Published:** 2008-01-19

**Authors:** Tomasz Kotwicki, Agata Walczak, Andrzej Szulc

**Affiliations:** 1 Department of Paediatric Orthopaedics and Traumatology, University of Medical Sciences, ul. 28 Czerwca nr 135, 61-545 Poznan, Poland

## Abstract

**Background:**

In patients with structural idiopathic scoliosis the body asymmetries involve the pelvis and the lower limbs; they are included in many theories debating the pathogenesis of idiopathic scoliosis.

**Methods:**

Hip joint range of motion was studied in 158 adolescent girls, aged 10–18 years (mean 14.2 ± 2.0) with structural idiopathic scoliosis of 20–83° of Cobb angle (mean 43.0° ± 14.5°) and compared to 57 controls, sex and age matched. Hip range of rotation was examined in prone position, the pelvis level controlled with an inclinometer; hip adduction was tested in five different positions.

**Results:**

In girls with structural scoliosis the symmetry of hip rotation was less frequent (p = 0.0047), the difference between left and right hip range of internal rotation was significantly higher (p = 0.0013), and the static rotational offset of the pelvis, calculated from the mid-points of rotation, revealed significantly greater (p = 0.0092) than in healthy controls. The detected asymmetries comprised no limitation of hip range of motion, but a transposition of the sector of motion, mainly towards internal rotation in one hip and external rotation in the opposite hip. The data failed to demonstrate the curve type, the Cobb angle, the angle of trunk rotation or the curve progression factor to be related to the hip joint asymmetrical range of motion.

**Conclusion:**

Numerous asymmetries around the hip were detected, most of them were expressed equally in scoliotics and in controls. Pathogenic implications concern producing a "torsional offset" of muscles patterns of activation around the spine in adolescent girls with structural idiopathic scoliosis during gait.

## Background

### Pathogenesis of idiopathic scoliosis

**Figure 1:**
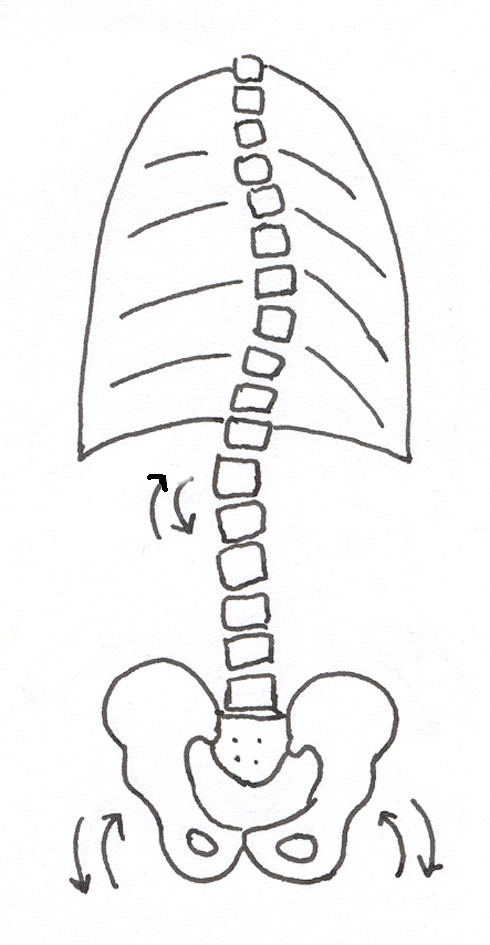
According to the Nottingham concept, in between the rotation inducing system and the rotation defending system, the mobile thoraco-lumbar vertebrae are susceptible to repeated rotation and tilt.

Idiopathic scoliosis (IS) is a three-dimensional deformity, which concerns not only the vertebral column, but the whole trunk, including the pelvis [1]. In the transverse plane, the scoliotic deformity is expressed by the axial rotation of vertebrae, with a rotational deformation of the trunk. The hypothetic origin of the deformation was postulated to raise either from the vertebrae [2] or the rib cage [3]. The resulting trunk deformity can be assessed clinically, with the use of an inclinometer, as proposed by Bunnell [4], or by Pruijs [5]. The most comprehensive theory of pathogenesis of idiopathic scoliosis, gathering together multiple anatomical or biomechanical causes and some premises, was proposed by Burwell et al., under the name of the Nottingham Concept [6], Figure 1. Hypothesis of a failure of control of cyclical rotations in the spine during gait ("dinner plate – flagpole mechanism") was formulated in this theory: the rotation-inducing system ("dinner plate") comprises "gait, femoral anteversion and the pelvis" [6], while the rotation-defending system ("flagpole") involves the rib cage and discs. The failure of rotation control of the spine "develops principally during gait due to asymmetrical forces resulting from rib-vertebra angle asymmetry" [6].

Body asymmetry in scoliotic subjects may involve the lower limbs, however there is a deficiency of studies documenting relation of lower limbs asymmetry to scoliosis. Cole et al. [7] found a significant correlation between hip ratios (external/internal rotation) and the Cobb angle. Saji et al. [8] examined frontal pelvis radiographs of scoliotic girls, and observed an increased but symmetrical neck-shaft femoral angle, however, considering the methodology of that study, an increased femoral anteversion would provide similar images. Burwell et al. [9] reported on a left-right ilio-femoral length asymmetry as well as bilateral anomalous lengthening of the tibia relative to the foot. Karski found a limitation of the right hip adduction, at the degree related to curve severity [10].

### Gait patterns in patients with scoliosis

Development of 3D gait analysis provided new findings concerning spatial motion of various body segments in gait. Methodology of these studies seems to be still under discussion. Usually three markers are placed on the pelvis (both anterior iliac spines and S1) and two markers at the shoulder girdle. Movements are assessed in relation to the line of progression (to the laboratory walls) or to other body segments. Mahaudens et al. [11] studied the spatial motion of the pelvis during gait in 12 patients with thoracolumbar or lumbar structural IS, and found no difference in the range of the 3D pelvis motion, comparing to 12 healthy controls. Although significant differences of the radiological morphology of the pelvis were found in scoliotics, comparing to healthy controls, these differences did not appear on gait analysis, with the exception of a 10.0% reduced step length. The authors postulated a prolonged abnormal duration of activation of the muscles of the back in scoliotic patients in gait. Chockalingam et al. [12] found a minimal change of the pelvic tilt and obliquity in gait, however, there was a variation of internal and external rotation of the pelvis, producing 4 to 10° left-right asymmetry. Giakas et al. [13] have not found the effect of the magnitude of scoliotic deformity on the scale of time and frequency domain of ground reaction forces. Kramers-de-Quervain et al. [14] analyzed the relative range of rotation motion between the pelvis and the shoulders, and found "a torsional offset" of the upper trunk in relation to symmetrically rotating pelvis. Their study involved 10 females aged 10 to 36 years, having the Cobb angle divergent from 9 to 73 degrees, with no control group. Crosbie and Vachalathiti suggested that consistent temporal interactions attributed to the synchrony of pelvic and hip joint motion during walking in normal subjects [15]. However, Nester [16] analyzed normal gait in healthy subjects to verify if the magnitudes of transverse rotations within the pelvis, the hips, the knees and the feet were interdependent; the author denied such a relation by registration of individual variations in the range of transverse plane motion of the hip, knee and the rearfoot.

The majority of the above cited findings apparently argue against the causative role of the rotation-inducing mechanism onto the spinal rotation. However, they do not seem to provide conclusive data. Several concerns are raised: (1) asymmetric mechanical influence of the pelvis on the spine is possible, even in case of symmetric pelvis motion in gait, by asymmetrical muscle activation, (2) gait laboratory analyses usually involve only several patients, presenting heterogeneous spinal deformities [11,14]. Lenke et al., describing scoliosis classification, regretted not having been in position to reduce the number of possible different radiological curve patterns to less than forty-two patterns [17]. (3) Reporting a segment kinematics in relation to the frame of reference: either the progression line, or the proximal body parts may lead to difficulty in data interpretation [16]. (4) The number and position of trunk markers are also discussed. Thus, the question, whether the "rotation inducing mechanism" exhibits symmetry in morphology and in motion, remains actual.

### Hip joint motion in scoliosis

**Figure 2:**
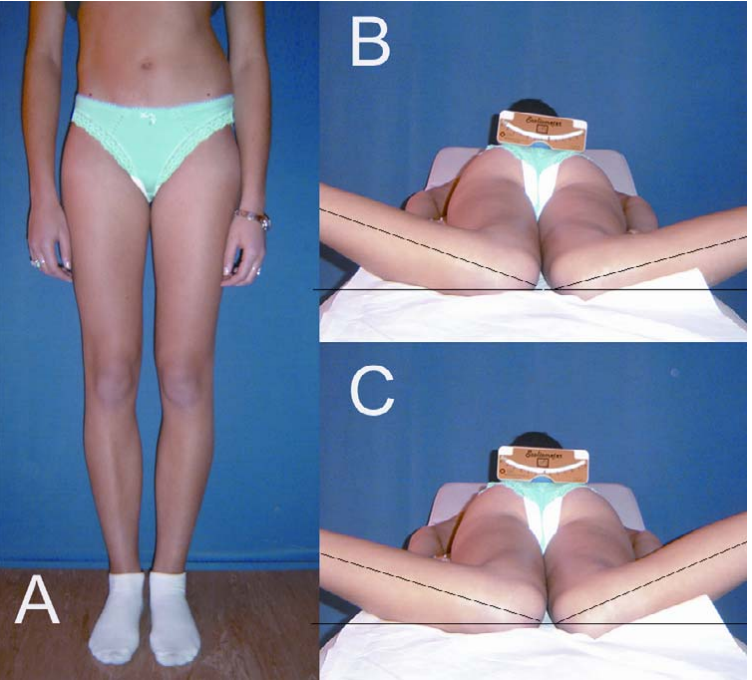
Asymmetrical pelvis, including both the position and the shape, is a common finding in children with idiopathic scoliosis (A). Apparent symmetry of hip internal rotation may be due to a non level pelvis (B). Scoliometer is a simple tool to enhance the precision of measures (C).

The hip joint is a ball-and-socket joint, which provides three-dimensional motion, containing antero-posterior, coronal and rotational components. In a standing or a supine subject, the hip joint is in neutral position, considered a zero starting position for testing the range of motion. For the scientific and didactic purposes the hip motion is assessed separately in each of the three planes: sagittal (flexion-extension), coronal (abduction-adduction) and transverse (internal rotation, IR – external rotation, ER). The normal range of internal or external rotation in children was reported to be 45 degrees [18], examination performed in prone position, the hip in neutral position (not in flexion), the knees in 90 degrees flexion, the legs serving as indicators of the angle of hip rotation. The pelvis should be level and motionless; this is controlled visually or with the examiner's hand [19]. When teaching medical students, we observed poor precision of such measures and an involuntary introduction of several degrees of rotation. This is why we applied an inclinometer to control the pelvis level in prone position [20], Figure 2. Once the scoliometer was started to be systematically used for hip joint range of rotation assessment, we began to observe hip asymmetry patterns in children with idiopathic scoliosis.

Comparing the results of the static physical examination of the range of hip rotation with the dynamic range of pelvis transverse motion obtained during gait analysis is not well documented for patients with scoliosis. However, this relation was extensively studied in cerebral palsy patients, who express a number of transverse plane static and dynamic pathologies in gait. The most reliable parameter to indicate the hip rotation in gait was found to be the mid-point of the passive range of hip rotation [21], calculated for each hip from the values of the passive internal and external rotations.

This study was imagined on the premise that the asymmetry of the hip joint range of motion implies an asymmetry of the pelvic motion during gait or asymmetrical effects above the pelvis. The hypothesis of the study was that the range of hip motion in children with idiopathic scoliosis is not symmetrical and there exists a relationship between the hip asymmetry and the spinal deformity.

The aims of the current study were:

1. To examine the range of hip joint motion in children with scoliosis using a particular technique designed to standardize pelvis position.

2. To analyze the distribution of hip joint motion asymmetries in children with scoliosis versus healthy subjects.

3. To investigate the hypothesized association between hip joint asymmetries and scoliosis patterns, curve magnitude or predisposition to curve progression.

## Materials and methods

One hundred and fifty-eight girls formed the study group. They all presented a structural idiopathic scoliosis. The Cobb angle of the main curvature was superior to 20 degrees, when measured at a standing frontal spinal radiograph, range 20.0° – 83.0°, mean 43.0° ± 14.5°. The age varied from 10 to 18 years, mean 14.2 ± 2.0 years. The girls reported to the clinic for a consultation with suspicion of scoliosis, or were addressed for brace treatment, or admitted to the department for scoliosis surgery. The control group comprised 57 girls from the school screening program, age matched. In the control group no radiography was made; idiopathic scoliosis was excluded, based on the clinical examination and on the value of the angle of trunk rotation inferior to 5 degrees, measure with the Bunnell scoliometer.

Cobb angle of the main curve was registered. Curves were classified according to a modified Lenke classification [17]: (1) structural single thoracic with non structural lumbar component (Lenke 1) – 57 girls, (2) structural single lumbar or thoracolumbar (Lenke 5) – 30 girls, (3) double structural curvatures: thoracic and lumbar or thoracic and thoracolumbar (Lenke 3 and Lenke 6) -71 girls. The other types (Lenke 2 double thoracic curves and Lenke 4 triple major) were excluded from the study. Curves superior to 25 degrees on standing radiographs were considered structural. Among 158 girls with scoliosis, two subgroups were created: (1) girls with progressive curvatures exceeding 50 degrees of Cobb angle (N = 53), and (2) post-menarchial girls with non-progressive curvatures not exceeding 30 degrees of Cobb angle (N = 49).

**Figure 3:**
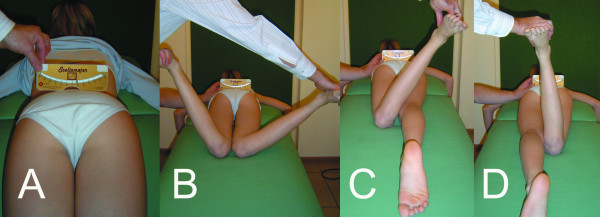
Technique of the measurement of the hip joint range of rotation. Patient in prone position, hips in neutral position, knees in 90° flexion. In spontaneous position there is an obliquity of the pelvis, masking asymmetry of rotation, and detected with the scoliometer placed over the posterior superior iliac spines (A). Asymmetry of the range of internal rotation: left hip = 25°, right hip = 55° (B). Asymmetry of the range of external rotation: left hip = 30° (C), right hip = 5° (D). The sum of rotation range is 55° for the left and 60° for the right hip. Transposition of the sector of rotation towards internal rotation may be diagnosed within the right hip, instead of claiming the limitation of hip motion. The mid-point of rotation is 2.5° for the left and 25° for the right hip. Therefore there is a static rotational offset of the pelvis of 22.5°, directed to the right side.

**Figure 4:**
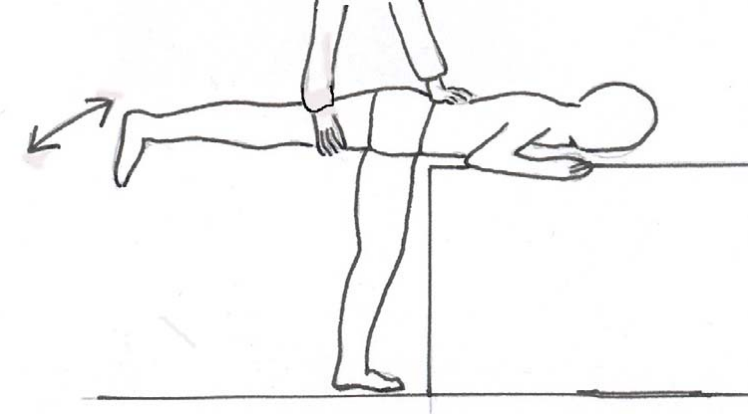
Range of hip adduction is precisely assessed in prone position, "at the end of the table", controlling the pelvis with one hand and the lower limb with the other hand.

The hip joint range of motion was tested: (1) in the prone position for internal and external rotation, and (2) in the supine, prone and lateral position for adduction. The patient was lying on a firm flat surface. The range of motion was assessed according to the standard criteria [19], in degrees of a circle with the hip joint in the centre of the circle, using a goniometer. The anatomic zero starting position was the intermediate between hip flexion and hip extension, between hip abduction and hip adduction, and between hip internal rotation and hip external rotation. The range of motion was determined in the passive range, with a goniometer, by one examiner (T.K.), and recorded to 5 degrees (5, 10, 15 etc.). To test the hip rotations in prone position, the examiner placed the Bunnell scoliometer over the sacrum, in the region of the posterior iliac spines, in order to provide a neutral pelvis position (zero of the scoliometer), Figure 3. The reproducibility was sufficient, controlled with the technical error of measurement calculated from ten measures by T.K. which amounted to 3 degrees. The hip joint range of adduction was tested in five different ways: in supine, lateral and prone position; in prone position the lower limb was kept in the neutral rotation, external or internal rotation, respectively (Figure 4).

The range of hip joint rotation was analyzed for: the left-right asymmetry, total range of hip rotation (the sum of external and internal range of rotation), correlation with the Cobb angle. The mid-point of the range of rotation was calculated, as proposed by Kerr et al. [21]. Then a static rotational offset of the pelvis (SROP) was calculated for each subject. The SROP was defined as the asymmetry of the mid-points (left and right) of rotation. In case of symmetry of the SROP equalled zero. All the measured parameters were compared with controls. Within the study group an internal analysis concerned comparison according to: (1) the curve pattern, (2) the Cobb angle of the main curve, (3) the Bunnell angle of trunk rotation of the main curve, (4) the progressive character of scoliosis.

## Results

### Study group

The range of internal rotation was 50.6 ± 13.4° (20.0 ÷ 90.0°) in the right hip, and 51.3 ± 13.5° (20.0 ÷ 90.0°) in the left hip; the range of external rotation was 35.2 ± 12.3° (5.0 ÷ 60.0°) in the right hip, and 34.8 ± 11.6° (5.0 ÷ 60.0°) in the left hip. The means and standard deviations for the whole group did not differ between the left and the right side, for both the IR and the ER (p > 0.05, Mann-Whitney test), Table 1. However, the symmetry of hip rotation was apparent. The IR was symmetrical only in 26 girls (16%), asymmetrical in 132 girls (84%): higher in the right hip in 63 girls and higher in the left hip in 69 girls. The ER was symmetrical in 45 girls (28%), asymmetrical in 113 girls (72%): higher in the right hip in 57 girls and higher in the left hip in 56 girls. Patient by patient calculation of the value of difference of the angle of IR between left and right hip revealed extremely significant difference (p < 0.0001, Wilcoxon matched-pairs signed-ranks test); a similar significant asymmetry between the left and right hip was found for the ER (p < 0.0001). This asymmetry was distributed as follows: an increased IR was combined with a decreased ER, while in the opposite side hip joint a decreased IR and an increased ER were observed (Figure 3). The correlation between IR and ER within one hip revealed significant negative correlation coefficient: -0.25 (p = 0.0018) for the right hip and -0.20 (p = 0.0108) for the left hip, Spearman rank correlation test. The correlation between the difference: IR_left _- ER_left _versus the difference: IR_right _- ER_right _was strong, the correlation coefficient r = -0.68, p < 0.0001, Spearman rank correlation test. The interpretation is that the advantage of the internal over the external range of rotation in one hip was combined with the opposite advantage (external over internal rotation) in the contra-lateral hip. The total range of hip rotation (the sum of the ranges of the IR and the ER) was symmetrical: 86.0 ± 15.6° (right hip) versus 86.2 ± 16.0° (left hip), difference not significant (p = 0.98, Mann-Whitney test). Totally, no limitation of the range of hip rotation was found, however the asymmetry in the distribution of the internal versus external range of rotation was demonstrated, accompanied with a mirror asymmetry within the opposite hip joint.

**Table 1:** Hip joint range of rotation in adolescent girls with scoliosis versus healthy controls.

Parameter	Scoliotics N = 158	Controls N = 57	P value
Internal rotation Right hip	50.6 ± 13.4 (20 ÷ 90)	52.8 ± 13.8 (25 ÷ 80)	NS
Internal rotation Left hip	51.3 ± 13.5 (20 ÷ 90)	52.7 ± 14.0 (30 ÷ 80)	NS
External rotation Right hip	35.2 ± 12.3 (5 ÷ 60)	37.9 ± 13.5 (10 ÷ 65)	NS
External rotation Left hip	34.8 ± 11.6 (5 ÷ 60)	37.5 ± 13.2 (10 ÷ 65)	NS
Total range rotation Right hip	86.0 ± 15.6 (50 ÷ 140)	88.0 ± 21.5 (30 ÷ 135)	NS
Total range rotation Left hip	86.2 ± 16.0 (60 ÷ 140)	90.3 ± 18.3 (50 ÷ 135)	NS
Difference in IR: Right minus left	-0.4 ± 9.9 (-30 ÷ 20)	0.1 ± 7.9 (-20 ÷ 20)	NS
Difference in ER: Right minus left	0.2 ± 9.3 (-25 ÷ 30)	0.4 ± 6.4 (-10 ÷ 20)	NS
Absolute difference left-right in IR	8.3 ± 5.4 (0 ÷ 30)	5.7 ± 5.4 (0 ÷ 20)	0.0013
Absolute difference left-right in ER	7.2 ± 5.9 (0 ÷ 30)	4.4 ± 4.6 (0 ÷ 20)	0.0023
Mid-point of rotation for right hip	7.8 ± 10.1 (-15 ÷ 38)	7.4 ± 10.4 (-20 ÷ 32.5)	NS
Mid-point of rotation for left hip	8.1 ± 9.6 (-15 ÷ 38)	7.6 ± 10.5 (-17.5 ÷ 32.5)	NS
Static rotational offset of the pelvis	7.0 ± 5.1 (0 ÷ 25)	5.0 ± 4.3 (0 ÷ 17.5)	0.0092

The means and standard deviations are given, followed by minimum and maximum in the brackets. IR – internal rotation, ER – external rotation. There is more important asymmetry in the range of hip rotation in girls with scoliosis than in controls, and in the severity of the static rotational offset of the pelvis.

### Control group

The range of internal rotation was 52.8 ± 13.8° (25.0 ÷ 80.0°) in the right hip and 52.7 ± 14.0° (30.0 ÷ 80.0°) in the left hip; the range of external rotation was 37.9 ± 13.5° (10.0 ÷ 65.0°) in the right hip and 37.5 ± 13.2° (10.0 ÷ 65.0°) in the left hip. The means and standard deviations did not differ significantly between the left and the right side for both the IR and the ER. Differences: range of the right hip rotation minus range of the left hip rotation were: 0.09 ± 7.9° (-20.0 ÷ 20.0°) for internal rotation and 0.35 ± 6.4° (-10.0 ÷ 20.0°) for external rotation. However, the symmetry once again was apparent: present only if the calculations were performed for the whole group. Case by case comparison of the range of rotation revealed marked asymmetry between left and right side. The IR was symmetrical in 20 girls (35%), asymmetrical in 37 girls (65%): higher in the right hip in 19 girls and higher in the left hip in 18 girls. The ER was symmetrical in 25 girls (44%), asymmetrical in 32 girls (56%): higher in the right hip in 17 girls and higher in the left hip in 15 girls. Case by case calculations of the left-right hip rotation asymmetry in the control group showed an extremely significant asymmetry (p < 0.001). The total range of hip rotation (the sum of the ranges of the IR and the ER) was symmetrical: 88.0 ± 21.5° (right hip) versus 90.3 ± 18.3° (left hip), difference not significant (p = 0.54, unpaired t-test), Table 1.

### The study group versus the control group

The mean values of the basic parameters concerning hip joint rotation did not differ between the study and the control group (Table 1). In the study group, fifty percent of the asymmetrical hips revealed superior range of rotation at the left side, while the other 50% on the right side; this concerned both IR and ER. The same proportion was found in the control group.

The proportion of the number of symmetric hips versus asymmetric hips was significantly different in the study group (26/132) versus the control group (20/37), p = 0.0047, Fisher's exact test. The hip rotation asymmetry was more frequent in girls with scoliosis. The absolute value of the difference between left and right hip range of rotation showed a significant difference of the means between scoliotics and controls in the range of asymmetry of the IR: 8.3 ± 5.4° versus 5.7 ± 5.4° (p = 0.0013, Mann-Whitney test), as well as of the ER: 7.2 ± 5.9° versus 4.4 ± 4.6° (p = 0.0023, Mann-Whitney test). The static rotational offset of the pelvis was present in 132 of 158 scoliotics and in 43 of 57 controls. The value of the SROP was significantly higher in scoliotic girls than in healthy controls (7.0 ± 5.1° versus 5.0 ± 4.3°, p = 0.0092, Mann-Whitney test), Table 1.

The subgroup with progressive curvatures (N = 53) did not demonstrate significant difference comparing to the subgroup with stable curvatures (N = 49) in any of the hip rotation derived parameters (Table 2). The prevalence of hips with symmetric rotations was lower in progressive curvatures than in stable ones (5 for 53 versus 11 for 49), but not significantly (p > 0.05).

**Table 2:** Comparison of the subgroup of progressive structural scoliosis with non-progressive structural scoliosis.

Parameter	Progressive N = 53	Stable N = 49	P value
Internal rotation Right hip	51.2 ± 15.1 (25 ÷ 90)	48.4 ± 12.1 (20 ÷ 75)	NS
Internal rotation Left hip	53.4 ± 15.1 (20 ÷ 90)	48.3 ± 11.5 (20 ÷ 70)	NS
External rotation Right hip	35.6 ± 12.9 (5 ÷ 60)	32.8 ± 11.1 (15 ÷ 60)	NS
External rotation Left hip	34.4 ± 10.9 (5 ÷ 50)	33.4 ± 12.0 (5 ÷ 60)	NS
Total range rotation Right hip	87.2 ± 17.7 (50 ÷ 140)	81.2 ± 12.8 (55 ÷ 110)	0.0549
Total range rotation Left hip	88.5 ± 16.4 (60 ÷ 130)	81.6 ± 11.7 (60 ÷ 110)	0.0563
Difference in IR: Right minus left	-1.2 ± 8.8 (-20 ÷ 15)	0.3 ± 10.3 (-30 ÷ 20)	NS
Difference in ER: Right minus left	1.1 ± 10.1 (-25 ÷ 20)	-0.5 ± 7.5 (-15 ÷ 20)	NS
Absolute difference left-right in IR	7.7 ± 4.5 (0 ÷ 20)	8.0 ± 6.3 (0 ÷ 30)	NS
Absolute difference left-right in ER	7.7 ± 6.6 (0 ÷ 25)	5.6 ± 4.9 (0 ÷ 20)	NS
Mid-point of rotation for right hip	7.9 ± 10.7 (-10 ÷ 37,5)	7.8 ± 9.8 (-15 ÷ 27.5)	NS
Mid-point of rotation for left hip	9.1 ± 10.1 (-15 ÷ 37,5)	7.4 ± 10.2 (-12.5 ÷ 32.5)	NS
Static rotational offset of the pelvis	7.0 ± 4.8 (0 ÷ 20)	6.4 ± 5.3 (0 ÷ 25)	NS

The means and standard deviations are given, followed by minimum and maximum in the brackets. IR – internal rotation, ER – external rotation. The values of the two subgroups do not differ significantly.

No differences in hip rotation derived parameters was found respective to the curve pattern, Table 3. There was no significant correlation between the parameters describing hip asymmetries (difference of the left and right IR or ER, static rotational offset of the pelvis) and the parameters describing the scoliosis: Cobb angle of the main curve, the Bunnell angle of trunk rotation of the main curve (p.0.05, Spearman nonparametric correlation).

**Table 3:** Hip joint range of rotation in adolescent girls with scoliosis depending on the curve pattern (Lenke classification).

Parameter	Lenke I N = 57	Lenke III and VI N = 71	Lenke V N = 30	P value
Internal rotation Right hip	50.4 ± 13.4 (25 ÷ 80)	51.4 ± 14.4 (20 ÷ 90)	50.2 ± 11.8 (30 ÷ 75)	NS
Internal rotation Left hip	48.9 ± 12.7 (20 ÷ 80)	52.9 ± 15.0 (20 ÷ 90)	52.4 ± 11.1 (30 ÷ 80)	NS
External rotation Right hip	34.5 ± 13.3 (5 ÷ 60)	35.2 ± 12.2 (10 ÷ 60)	35.2 ± 12.2 (20 ÷ 60)	NS
External rotation Left hip	35.1 ± 10.4 (15 ÷ 60)	35.1 ± 12.7 (5 ÷ 60)	34.6 ± 11.4 (10 ÷ 60)	NS
Total range rotation Right hip	84.7 ± 15.0 (55 ÷ 130)	87.1 ± 17.1 (50 ÷ 140)	87.1 ± 13.6 (65 ÷ 120)	NS
Total range rotation Left hip	84.2 ± 14.3 (60 ÷ 125)	88.2 ± 17.4 (60 ÷ 140)	87.1 ± 15.7 (60 ÷ 130)	NS
Difference in IR: Right minus left	1.5 ± 9.8 (-15 ÷ 20)	-1.4 ± 9.8 (-30 ÷ 20)	-2.1 ± 9.8 (-15 ÷ 15)	NS
Difference in ER: Right minus left	-0.5 ± 9.7 (-20 ÷ 20)	0.1 ± 10.1 (-25 ÷ 30)	1.2 ± 6.5 (-15 ÷ 10)	NS
Absolute difference left-right in IR	8.6 ± 4.7 (0 ÷ 20)	7.6 ± 6.3 (0 ÷ 30)	9.2 ± 4.2 (0 ÷ 15)	NS
Absolute difference left-right in ER	8.0 ± 5.4 (0 ÷ 20)	7.7 ± 6.5 (0 ÷ 30)	4.5 ± 4.9 (0 ÷ 15)	0.0194
Mid-point of rotation for right hip	8.0 ± 11.0 (-10 ÷ 37,5)	8.1 ± 10.1 (-15 ÷ 27.5)	6.6 ± 9.4 (-10 ÷ 27.5)	NS
Mid-point of rotation for left hip	6.6 ± 8.9 (-12,5 ÷ 25)	8.8 ± 10.8 (-15 ÷ 37.5)	8.9 ± 8.0 (-7.5 ÷ 25)	NS
Static rotational offset of the pelvis	7.6 ± 4.8 (0 ÷ 20)	6.8 ± 5.9 (0 ÷ 25)	6.0 ± 3.8 (0 ÷ 12.5)	NS

The means and standard deviations are given, followed by minimum and maximum in the brackets. IR – internal rotation, ER – external rotation. One way ANOVA was used for normal distribution, Kruskal-Wallis test for not normal. The values of the three columns do not differ significantly, apart from the absolute difference of the range of external rotation, signifying less asymmetry in Lenke V curves.

No significant difference was found in the proportion of symmetric/asymmetric hip adduction between patients with scoliosis and controls, irrespective the technique of examination (Table 4).

**Table 4:** The hip adduction range of motion in the study and control groups, analyzed as symmetric or asymmetric range between the left and the right hip.

Testing position	Scoliosis N = 46		Controls N = 46		X^2 ^test p value
		
	Symmetry	Asymmetry	Symmetry	Asymmetry	
Supine	20	26	18	28	0.67
Lateral	21	25	25	21	0.40
Prone 0	24	22	27	19	0.53
Prone Ext	25	21	30	16	0.29
Prone Int	22	24	29	17	0.14

Prone 0 – examination in the prone position with the lower limb in neutral rotation; Prone Ext – examination with the lower limb in external rotation; Prone Int – examination with the lower limb in internal rotation. No significant difference was found in the proportion of symmetric/asymmetric hip adduction between patients with scoliosis and the controls, irrespective the technique of examination.

## Discussion

### Using inclinometer to enhance precision of hip motion measures

The idea of the use of the scoliometer to standardize pelvis position raised from the authors' observations, that medical students presented obvious difficulties in assessing the range of hip motion, even helped with a goniometer. Usually, the pelvis was not correctly stabilized, so it moved together with the moving lower limb. Moreover, the neutral pelvis rotation was erroneously assessed. We solved both technical pitfalls with the use of the scoliometer. We consider the classical way of pelvis stabilization (with one examiner's hand) very useful to detect the moment the pelvis begins to tilt or rotate. However, the neutral position of the pelvis cannot be precisely controlled, unless the scoliometer is used. Some technical points may be raised: (1) Shortening of the rectus femoris muscle may be responsible for elevation of a hemipelvis during knee flexion; we recommend performing the clinical test of Ely in order to detect rectus femoris shortening. (2) In the patient lying prone, the pelvis is not level spontaneously, usually the pelvis presents some degree of rotation, which is expressed by elevation of one side. (3) The iliac bones are susceptible to nutation and counter-nutation movements in the iliosacral junction, creating asymmetric position of the iliac spines. Thus, a part of the asymmetry found in this study could be attributed to the intrinsic pelvic deformation.

### Hip joint asymmetry in patients with idiopathic scoliosis

The range of motion of the joints is normally symmetrical, and this serves in clinical practice to detect the affected limb, by comparing with the "healthy" limb. Generally, the amplitude of joint motion decreases in the presence of a pathology of the joints, bones or muscles. In our study, the global amplitude of the hip rotation motion, calculated as the sum of the internal rotation range and external rotation range, revealed no limitation. Thus, the traditional term of pathology seems not justified to describe the asymmetries found both in girls with scoliosis and in healthy controls. However, there was a significant asymmetry between left and right hip joint in the corresponding movements, namely medial rotation, lateral rotation, and adduction. We propose to apply the term of transposition of the sector of the hip joint motion.

Our results document numerous asymmetries around the pelvis in adolescent girls with idiopathic scoliosis. Both the prevalence of the hip asymmetry and its severity significantly exceeded the values of the control group of healthy adolescents sex and age matched. The range of left-right hip rotation asymmetry was higher in scoliotic girls comparing to controls; the difference being statistically significant. The left – right hip asymmetry of the range of rotation was extremely significant (as in the controls either). There was a pitfall to compare means, which were equal for the left and right side. Saji et al. [8] did not observed any left-right asymmetry of the femoral neck-shaft angle in patients with scoliosis, however their methodology comprised comparing the means, while calculating the absolute value of the left-right differences for each patient was not performed. In our study, only in 26 of 158 girls with scoliosis the range of rotation was symmetrical, in the remaining 132 girls the asymmetry ranged from 5 to 30 degrees. Increased internal rotation was usually accompanied by decreased external rotation and vice versa; the total range of rotation movement being the same. Thus, a static rotational offset of the pelvis was demonstrated.

Our results failed to demonstrate a significant difference in the values of the parameters of the hip joints range of rotation between the progressive and the non-progressive scoliosis (Table 2). Also, no relation of hip asymmetries to the radiological Lenke curve classification was found (Table 3).

In this study, the asymmetric range of hip rotation was documented in the cross-sectional pattern, with no longitudinal evaluation. This seems justified, unless the hip asymmetries are considered secondary to spinal curvatures. Thus, the evolution in the range of hip motion should be studied over time.

A specific asymmetry of the hip joint range of motion, namely the limitation of the adduction of the right hip, was hypothesized to be an etiologic factor for idiopathic scoliosis [10]. Our findings regarding the right hip adduction in children with scoliosis did not follow the results reported by Karski. For a better precision, we checked the hip adduction in three positions: supine, lateral and prone. Our impression was that the prone position "at the end of the table" provided the best conditions for measurements, because: (1) the patient does not need to change the position during exam, (2) the pelvis is easily controlled with one hand while the other hand controls the flexion and rotation of the thigh.

The interpretation of our findings can be made not only under the Nottingham concept. Hip joint static asymmetries could be the consequence of muscle imbalance (primary or secondary), or of morphological asymmetries of the pelvis. Rigo [22] described an indirect radiological factor (BSIIa difference: bisacro-iliac-ischial angle difference) to assess the pelvis torsion. With this factor, the author was able to make the difference between the iliac rotation asymmetry (due to nutation/counter-nutation movements) and the structural intra-bone iliac deformity. He also combined patterns of pelvis asymmetry with structural changes inside the lumbar spine (wedging in L3/L4 disc). The author concluded that the pelvis asymmetries could be considered as a part of the whole body torsional phenomenon rather than cause-effect factors. Considering the fact that in the current study the asymmetries were observed in a significant number of normal subjects just with a lower prevalence and degree, our findings could be also interpreted following the theory of idiopathic scoliosis as a sign of developmental instability [23], where scoliotic subjects present a major degree of directional and fluctuating asymmetry [24], as a factor indicating their predisposition to develop scoliosis.

### Implications for gait in scoliotics

The static rotational offset of the pelvis revealed more important values in girls with scoliosis, than in healthy adolescents. That means the pelvis is not as balanced in the transverse plane, as in controls. However, the difference seems moderate, and it was difficult for us to attribute a particular physiological meaning to this finding.

Debating the biomechanical factors of pathogenesis of idiopathic scoliosis, our findings correspond to the Nottingham concept. We found a significant asymmetry in the internal/external rotation between both hips, which might be the origin of the asymmetry of the "dinner plate mechanism". Kramers-de-Quervain [14] reported asymmetric rotation of the trunk during gait in between symmetrical rotation of the pelvis and the head. The magnitude of the torsional offset correlated with the degree of the thoracic component of the scoliotic deformity. Burwell et al.[6]  found a more important asymmetry of femoral anteversion in school screening referrals than in control subjects. Giakas et al [13] constructed the study to reveal eventual overloading of the lower limbs during gait due to the bending of the spine in one side. The authors failed to demonstrate such an effect and suspect that subjects with scoliosis have an unidentified functional difference that acts to prevent asymmetric loading of the ground reaction forces during gait.

There is a tendency to optimize the economy of the muscle effort in gait. Symmetrical motion of the pelvis is most economic; an abnormal pelvis swing provokes more important muscle effort. In scoliotic subjects the non ergonomic movements of the pelvis resulting from hip asymmetries usually are not observed, probably being corrected by asymmetric activation of back and lower limbs muscles. On the other hand, keeping a symmetric position of the head in gait is an obviousness. Thus, the effective walking function implies that the thoracic and lumbar spinal segments stay mobile in between actively stabilized upper and lower trunk, so they may be susceptible to asymmetric position/motion. Muscle activation pattern necessary to maintain the ergonomic gait may result in abnormal activation of trunk muscles, with subsequent asymmetric arms of force of the muscles attached to the vertebral column. Thus, the rotation-inducing mechanisms (mechanical component of the Nottingham concept) may provide an asymmetric action on the spine, even if they reveal a symmetrical motion in gait.

Does the "dinner plate" turns asymmetrically? Our study demonstrated a significantly higher static rotational offset of the pelvis in adolescent girls with idiopathic scoliosis comparing to age matched controls. During gait the 3D pelvis tilts are supposed to be actively minimized for a better gait ergonomics, the dynamic stabilization of the static pelvis rotational offset may be a source of abnormal (asymmetric) activation of back muscles. This provides arguments that the "dinner plate" may have an asymmetric influence on the spine, in spite it moves symmetrically during gait, by producing a "torsional offset" of muscle forces around the spine, a mechanism potentially contributive to scoliosis development/progression.

## Conclusion

Symmetry of the range of hip joint rotation was found only in 16% of patients with scoliosis and in 35% of healthy controls. The remaining subjects presented various patterns of asymmetry around the hip joints, some of this asymmetries were more frequent and more severe in adolescent girls with progressive IS, than in controls. In girls with structural scoliosis the symmetry of hip rotation was less frequent (p = 0.0047), the difference between left and right hip range of rotation was significantly higher (p = 0.0013), and the static rotational offset of the pelvis, calculated from the mid-points of rotation, revealed significantly greater (p = 0.0092) than in healthy controls. The detected asymmetries comprised no limitation of hip range of motion, but a transposition of the sector of motion, mainly towards internal rotation in one hip and external rotation in the opposite hip. Thus, a static rotational offset of the pelvis, significantly greater in adolescents with idiopathic scoliosis than in controls, was demonstrated. The use of the scoliometer revealed advantageous for detection of mild and moderate asymmetries of the hip joints range of rotation. The initial hypothesis on the association of the hip asymmetrical range of motion with the spinal deformity could not be confirmed. The data failed to demonstrate the curve type, the curve magnitude or the curve progression factor to be related to the hip joint asymmetrical range of motion. Pathogenic implications concern producing a "torsional offset" of muscles patterns of activation around the spine in adolescent girls with structural idiopathic scoliosis during gait.

## Competing interests

The author(s) declare that they have no competing interests.

## Authors' contributions

T.K. – study design, collecting data, data analysis and interpretation, manuscript drafting

A.W. – collecting data, references search

A.S. – data interpretation, manuscript revision

All authors have read and approved the final manuscript.
